# Microwave-enhanced photocatalysis on CdS quantum dots - Evidence of acceleration of photoinduced electron transfer

**DOI:** 10.1038/srep11308

**Published:** 2015-06-17

**Authors:** Fuminao Kishimoto, Takashi Imai, Satoshi Fujii, Dai Mochizuki, Masato M. Maitani, Eiichi Suzuki, Yuji Wada

**Affiliations:** 1 Department of Applied Chemistry, Tokyo Institute of Technology, 2-12-10-E4-3 Ookayama, Meguro, Tokyo 152-8551, Japan; 2 Knowledge-Intensive Collaborative Research Center, Chiba University, 1-33 Yayoi-cho, Inage-ku, Chiba 263-8522, Japan

## Abstract

The rate of electron transfer is critical in determining the efficiency of photoenergy conversion systems and is controlled by changing the relative energy gap of components, their geometries, or surroundings. However, the rate of electron transfer has not been controlled by the remote input of an external field without changing the geometries or materials of the systems. We demonstrate here that an applied microwave field can enhance the photocatalytic reduction of bipyridinium ion using CdS quantum dots (QDs) by accelerating electron transfer. Analysis of the time-resolved emission decay profiles of CdS quantum dots immersed in aqueous solutions of bipyridinium exhibited the shortening of their emission lifetimes, because of the accelerated electron transfer from QDs to bipyridinium under microwave irradiation. This discovery leads us to a new methodology using microwaves as an external field to enhance photocatalytic reactions.

Electron transfer is the key for increasing the efficiency of photocatalysis and dye-sensitized solar cells (DSSCs)^1^,^2^. The rate of electron transfer is controlled by changing the relative energy gap of components^3^, their geometries^4^ or surroundings^5^, but has not been controlled by the remote input of the external field without changing the geometries or materials in the systems. Focusing on photocatalysis and DSSCs, the electron transfer between an organic moiety and the surface of an inorganic semiconductor plays a key role in charge separation, determining the final efficiencies of the photocatalytic reactions and the photoenergy conversion efficiency of DSSCs. Many studies have been performed to control the behavior of the excited electrons generated by the photon absorption in photocatalysis and DSSCs. For example, the rapid transfer of an excited electron followed by the absorption of photons is important for efficient charge separation in photocatalysis^4^,^5^. The excited electrons and the simultaneously generated holes are transferred to the reaction sites for reducing the electron acceptor molecules and oxidizing the donor molecules, respectively^6^. In DSSCs, the excited dye injects a hot electron into the conduction band of TiO_2_, which is immediately transferred to the transparent conductive layer^7^.

Effects of various microwave on chemical reactions have been observed, reported, and compared with those reactions performed using conventional heating. These effects are classified into thermal effects^8^,^9^,^10^,^11^ and non-thermal effects^12^,^13^. Microwave thermal effects can be realized by applying the characteristics of microwave heating such as rapid heating^8^,^9^ and substance-selective heating^10^,^11^ and are attributed to the mechanism of microwave heating in which the alternating electromagnetic fields interact with substances. However, the non-thermal effects are yet to be studied in the appropriate manners and need to be investigated to clarify their mechanism. Microwave non-thermal effects have been extensively studied in the organic synthesis field and in reactions at solid surfaces in several systems. Horikoshi *et al*.^13^ showed that a photodegradation reaction of a Rhodamine B organic dye with a TiO_2_ photocatalyst was enhanced under microwave irradiation, and this observation was attributed to microwave non-thermal effects on photocatalytic redox reactions. Recently, our group reported^14^ that the dechlorination reactions of an organohalide by Fe particles were accelerated under microwave heating compared with those reactions performed under conventional heating. These observations of the enhanced reaction rates under microwaves have led us to a hypothesis that the electron transfer reaction occurring at the interface between a solid surface and a liquid phase is accelerated by microwaves, which may be the origin of the microwave non-thermal effect observed in redox reactions. This study exhibits a direct evidence of microwave non-thermal effects observed as the acceleration of electron transfer.

## Results

**Figure 1:**
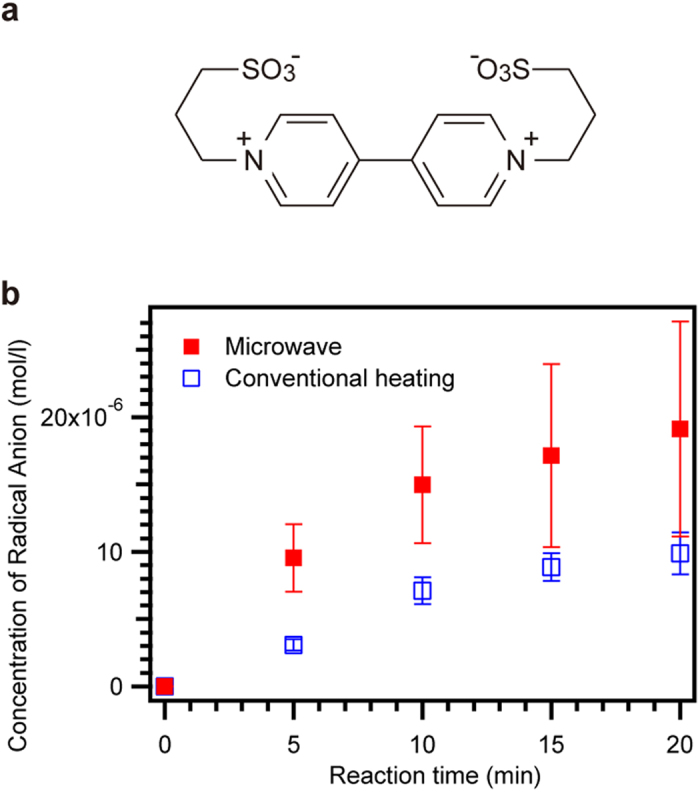
Photocatalytic reduction of PVS by CdS QDs **(a)** Chemical structure of N,N’-bis(3-sulfonatopropyl)-4,4′-bipyridinium (PVS). **(b)** Time variation plots of the PVS radical anion concentration under microwaves at 1.5 W and conventional heating. The reaction mixture contains 2 mM PVS, H_2_O, MeOH, and the CdS QDs anchored on SiO_2_ particles. The reaction temperature was 52 °C. The error bars were estimated by standard deviation of three experiments.

First, we attempted to observe the enhanced photocatalytic reduction of bipyridinium by CdS quantum dots under microwave irradiation. We selected N, N’-bis(3-sulfonatopropyl)-4, 4′-bipyridinium (PVS^15^, Fig. 1a) as an electron acceptor, and CdS quantum dots (QDs) anchored on SiO_2_ particles as a photocatalyst. Because the PVS anion radical generated by photoreduction absorbs visible light, the concentration of anion radicals was determined using UV-Vis spectroscopy. The UV-Vis absorption spectra are shown in Extended data Fig. 1. Peaks at 602 nm are attributed to the PVS radical anion. Fig. 1b shows the time variation plots of the anion radical concentrations under microwaves and conventional heating. The temperature of both systems was kept at 52 °C. The generation rate of the anion radicals under microwaves was two times faster than that under conventional heating, exhibiting the enhancement of the photocatalytic activity of CdS QDs. Large error bars were observed in the latter stage of the microwave experiments, whereas the error bars were smaller while conventional heating. Because SiO_2_ particles with anchored CdS QDs were dispersed in the reaction solution by ultrasonication before starting the reaction and the solutions were not stirred during the experiments, they were sinking during the reaction time. The scattering caused by dispersed SiO_2_ particles was reflected as a strong background in the absorption spectra, as shown in Extended data Fig. 1a,c, and was lowered with the reaction time because of the sinking of SiO_2_ particles. The error bars were estimated as the standard deviation from three experiments. Sinking SiO_2_ particles with anchored CdS QDs in the solution may cause the unstable distribution of the microwave’s electromagnetic field, probably giving larger errors in the microwaves experiments than the conventional heating ones.

**Figure 2:**
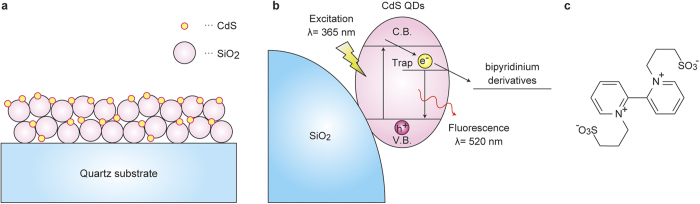
Structure of CdS QDs/SiO_2_ thin films and electron transfer scheme. (**a**) Structure of CdS QDs/SiO_2_ thin films. The average diameters of SiO_2_ particles and CdS QDs were 20 nm and 2.4 nm, respectively. (**b**) Energy diagram of the electron transfer process from CdS QDs to bipyridinium derivatives. (**c**) Chemical structure of N,N’-bis(3-sulfonatopropyl)-2,2′-bipyridinium (DQS).

Hereafter, our interest focused on the effect of microwaves on the electron transfer from excited CdS QDs to bipyridinium ions, which is one elementary process of the whole photocatalytic reaction, comprising the adsorption of the substrate, photoabsorption, injection of the excited electron into the substrate, and desorption of the reduced substrate. We planned to observe the electron transfer distinguished from the other elementary steps using the accurate measurement of the reaction temperatures proposed by Kappe *et al*.^16^. Consequently, we selected time-resolved photoemission decay measurements of CdS QDs occurring in a sub-nanosecond order, which allow for the evaluation of the electron transfer rates from photoexcited CdS QDs adsorbed on SiO_2_ nanoparticle thin films (CdS QDs/SiO_2_ thin films; Fig. 2a) to liquid phase bipyridinium derivatives (Fig. 2b). The relaxation processes of the photoexcited state of CdS QDs can be accurately analyzed by measuring the time-resolved photoemission of CdS QDs. Furthermore, the adsorption and desorption processes can be excluded, and only the electron transfer process is studied using time-resolved photoemission decay measurements because the time constants of the adsorption and desorption of the electron acceptor molecules are extremely slow compared with those of electron transfer.

A previous report suggested that an electron transfer reaction from the QDs surface defect level to organic molecules is faster than that from the QDs conduction band^17^. We obtained no experimental results to support that the electron transfer from the QDs surface defect level to organic molecules is faster than that from the QDs conduction band in the present study. However, we observed the behavior of the majority of electrons, which is reflected as fast electron transfer. Furthermore, the longer the lifetime of the excited electron, the more observable are the effects of microwaves. The lifetime of the excited electron in the conduction band was reported to be much shorter than that in the defect level^18^. Finally, we selected the photoemission at 520 nm (excitation of 365 nm), which is attributed to the photoemission process from a surface defect level of CdS QDs for the analysis of time-resolved emission decay spectra. Temperature measurements were performed using a fiber-optic thermometer (Opsens, Picosens) installed in close proximity to the sample. The input power generating the microwaves was 1.0 W. The emission decay measurements were initiated when the temperature of the solution containing the immersed samples reached 52 °C. The temperature setting under conventional heating experiments performed as a control measure was also kept at 52 °C. The temperature profiles under microwaves and conventional heating during the time-resolved emission decay measurements are shown in Extended data Fig. 2, indicating that the same temperatures were retained for both experiments.

**Figure 3:**
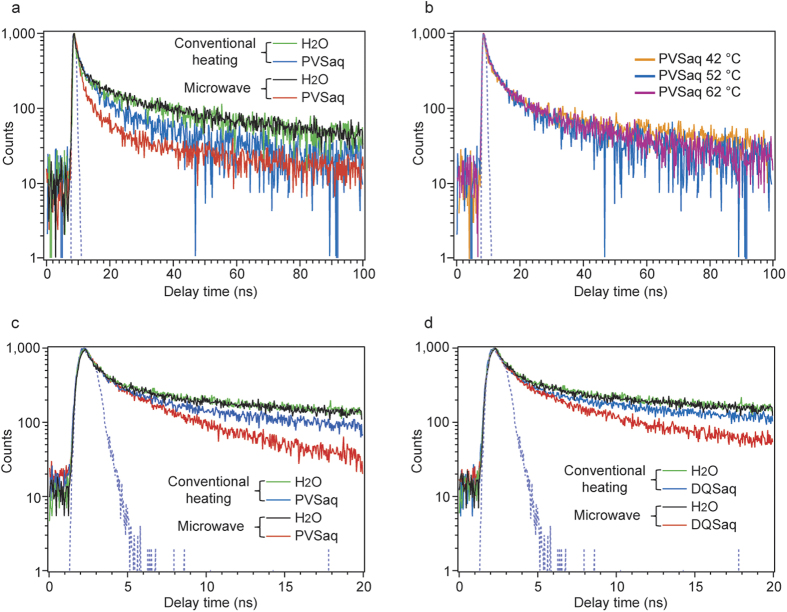
Measured time-resolved emission decay profiles. (**a**) Time-resolved emission decay profiles of CdS QDs immersed in deionized water or PVS aqueous solution under microwave irradiation or conventional heating (time range = 100 ns). Blue dotted spectrum was the instrument response function. (**b**) Temperature dependence of time-resolved emission decay profiles of CdS QDs immersed in a PVS aqueous solution. These were measured at different solution temperatures: 42 °C, 52 °C, and 62 °C. (**c**) Time-resolved emission decay profiles of CdS QDs immersed in deionized water or PVS aqueous solution under microwave irradiation or conventional heating (time range = 20 ns). (**d**) Time-resolved emission decay profiles of CdS QDs immersed in DQS aqueous solution under microwave irradiation or conventional heating (time range = 20 ns).

The time-resolved emission decay profiles of CdS QDs/SiO_2_ thin films immersed in water or PVS aqueous solution are shown in Fig. 3a. The emission lifetime of CdS QDs/SiO_2_ thin films immersed in PVS solution was clearly shortened under microwave irradiation, whereas no change in water is seen under microwave irradiation. The emission decay was measured three times (Extended data Fig. 3). Because the profile in water without PVS under conventional heating is the same as that under microwave irradiation, the luminescence and non-luminescence processes in CdS QDs were unaffected by microwaves. The time-resolved emission decay profiles of CdS QDs/SiO_2_ thin films immersed in PVS solution under conventional heating at 42 °C, 52 °C, and 62 °C (Fig. 3b) exhibited no significant change on temperature alteration. Therefore, we suggest that the change in emission lifetime under microwave irradiation was not caused by “nonequilibrium local heating,” as reported by Tsukahara *et al*. for Co particles dispersed in dimethyl sulfoxide under microwave irradiation^11^. Furthermore, the result that the temperature did not affect the time-resolved emission decay profiles strongly supports that the adsorption–desorption processes can be excluded from the analyses performed in this study. Data led us to conclude that the shortening of the lifetime is not because of the changes in temperatures, i.e., the microwave thermal effects, but because of the microwave non-thermal effects shown by the acceleration of excited electron transfer from CdS QDs to PVS.

The emission lifetimes and electron transfer rates were calculated by fitting the time-resolved emission decay profiles. Table 1 shows the fitting analysis results of the time-resolved emission decay profiles shown in Fig. 3a. All fitting results are shown in Extended data table 1. When the profiles of CdS QDs in water were fitted by a biexponential function, the τ values and relative amplitudes, *A*_*Normalized*_, under microwave irradiation are nearly identical to those under conventional heating. The τ_1_ and τ_2_ values were ca. 2 ns and 35 ns, respectively. The shorter lifetime, τ_1_, was attributed to the reassociation process of the surface defect level electrons effectively relaxing from the conduction band, whereas the longer lifetime, τ_2_, was assigned to the reassociation process of electrons that diffuse in the crystal lattice before relaxing into the defect level^18^. In contrast, the time-resolved emission decay profiles of CdS QDs in PVS solution were fitted by a triexponential function because the chi-square values were large when the decay profiles were fitted by a biexponential function (see Extended data table 2). The longest lifetimes under microwave irradiation and conventional heating were almost the same, ~35 ns. Because the longest lifetime value in PVS solution, τ_3_, was equal to the longest value in water, this lifetime was attributed to the same emission process. The two shorter lifetimes, τ_1_ and τ_2_, that appeared in the time-resolved emission decay profiles of CdS QDs in PVS solution were attributed to the electron transfer from CdS QDs to PVS and are the processes that only exist in the presence of PVS. The measured time range was shortened to 20 ns from 100 ns to precisely determine the lifetimes of τ_1_ and τ_2_.

To examine the components with short lifetimes, the emission decays of CdS QDs in water, PVS aqueous solution, or DQS (N,N’-bis(3-sulfonatopropyl)-2,2′-bipyridinium^19^, Fig. 2c) were measured within a 20 ns time range (Fig. 3c,d). The emission decay was measured three times. (Extended data Fig. 4) The reduction potential of DQS (−0.75 V vs. NHE) is more negative than that of PVS (−0.41 V vs. NHE). Because the decay measured in the time range of 20 ns partially included the process with a lifetime of 35 ns in the latter time stage of the measurement, τ_2_ was fixed as 35 ns when the time profile of CdS QDs in water were fitted by a biexponential function, and τ_3_ was fixed as 35 ns when the time profiles of CdS QDs in PVS solution or DQS solution were fitted by a triexponential function (table 2). All fitting results are shown in Extended data table 3. From the fitting results, the average lifetime, τ_av_, was calculated using the following equation:


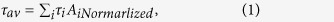


where τ_*i*_ and *A*_*iNormalized*_ are fluorescence lifetime and relative amplitudes of *i*th component, respectively. Using the τ_av_ value, the electron transfer reaction rate, *k*_*ET*_, was determined by the Stern–Volmer equation:


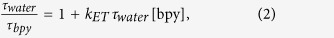


where τ_Water_ and τ_bpy_ are the average lifetimes of CdS QDs immersed in water and PVS or DQS aqueous solutions, respectively, and [bpy] is the PVS or DQS concentration, 2 mM. The Stern–Volmer equation was used for calculating the electron transfer rate under the supposition that the reaction mechanisms are the same under microwave and conventional heating. The obtained electron transfer rates from CdS QDs to PVS under microwave irradiation and conventional heating were 2.6 × 10^11^ M^−1^s^−1^ and 1.0 ×  10^11^ M^−1^s^−1^, respectively. However, the electron transfer rates from CdS QDs to DQS under microwave irradiation and conventional heating were estimated as 2.4 × 10^11^ M^−1^s^−1^ and 6.8 × 10^10^ M^−1^s^−1^, respectively. These values of the electron transfer rates clearly demonstrate the acceleration of the transfer of the excited electron generated from CdS QDs to bipyridinium derivatives.

We emphasize that no acceleration of the electron transfer was observed for the dispersion of CdS QD in the presence of methyl viologens under microwaves. Extended data Fig. 5 exhibits the time profiles of the photoemission decay of the freely dispersed CdS QDs for microwave irradiation and conventional heating. A physical insight to an origin of the acceleration of the transfer of the excited electron is discussed below.

## Discussion

The Marcus theory of electron transfer reactions^20^ is employed to discuss the acceleration of electron transfer observed in the present study. This theory can be applied to asymmetric non-adiabatic electron transfer reactions between semiconductor crystals and organic molecules^21^. According to Marcus theory, electron transfer rates depend on the electronic coupling matrix element related to the electron orbital overlap of the donors and acceptors (*H*_*AB*_^2^) and the reorganization energy (λ), which is described as follows:


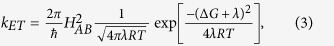


where ℏ is the reduced Planck’s constant, *R* is gas constant, *T* is the reaction temperature, and *ΔG* is Gibbs free energy change.

We assume that the electron transfer occurs from only the CdS QDs surface defect level. The potential of the surface defect level corresponding to the 520 nm emission is approximately −0.8 V vs. NHE^22^. Then, we can estimate the two important values of *H*_*AB*_^2^ and λ for comparing microwaves and conventional heating. The *H*_*AB*_^2^ values under microwave irradiation and conventional heating were estimated by equation (3) as 3.8 × 10^−26^ kJ^2^ mol^−2^ and 2.0 × 10^−26^ kJ^2^ mol^−2^, respectively, whereas the reorganization energies were estimated as 21.5 kJ mol^−1^ under microwave irradiation and 22.8 kJ mol^−1^ under conventional heating. By using microwave irradiation, the *H*_*AB*_^2^ value increases more than double, whereas the reorganization energies are approximately the same under microwave irradiation and conventional heating. Therefore, the acceleration of electron transfer observed in the present study can only be attributed to the change in *H*_*AB*_^2^ values. As for the mechanisms of microwave non-thermal effects, we raise an explanation of the accelerated electron transfer using the collective vibration of electron clouds in the CdS conduction band induced by microwaves, which is similar to the action of local surface plasmon resonance observed in metal nanocrystals^23^. Furthermore we cannot exclude other explanations such as Maxwell–Wagner polarization effects at the interface between CdS quantum dots and their surroundings (electrolyte or surface-modified molecules on CdS), and the promotion of electron mobility in CdS conduction bands induced by the alternating electromagnetic field. Such mechanisms were hypothetically applied to explain the measurements of the charge-carrier concentrations in semiconductors^24^.

In conclusion, we reveal that the photocatalytic reduction of bipyridinium ion using CdS QDs is enhanced by microwaves. The determination of the electron transfer rates from CdS QDs to bipyridinium derivatives (PVS or DQS) under microwave irradiation or conventional heating using time-resolved emission decay measurements demonstrates that the electron transfer rate under microwave irradiation is higher than that under conventional heating. The acceleration of electron transfer can be explained by the collective vibration of electrons by microwaves. These observations provide a new aspect to understanding microwave non-thermal effects, which was previously unknown, leading to systematically extending new chemistry using externally applied electromagnetic fields.

## Methods

### Synthesis of CdS QDs

We synthesized CdS QDs with a diameter of ~2.4 nm by a procedure adapted from Ref. 25. We heated a mixture of 12.8 mg of CdO (Sigma-Aldrich), 0.095 mL (85 mg) of 1-octadecene (Sigma-Aldrich), and 4.9 mL (3.9 g) of oleic acid (Wako Chemicals) to 300 °C under nitrogen. Sulfur (1.6 mg) dissolved in 1-octadecene (2.53 ml, 0.08 wt%) was injected into the above solution and stirred for 300 s. The resultant solution was cooled to room temperature. Unreacted CdO was removed from the solution by extraction with a mixture of methanol and chloroform. The QDs were obtained as a precipitate by adding acetone and separated by centrifugation and decantation. The resultant powder was re-suspended in dichloromethane or toluene to prepare the dispersion solution. This solution was filtered by a syringe filter to remove the residues, giving a clear colloidal solution. The obtained CdS QDs were covered by a uniform organic layer of oleic acid. The layer has a role for suppressing the aggregation of the particles and as a tunneling barrier of the photoinduced electron transfer^26^. This tunneling barrier might be involved as one of the barriers in the whole electron transfer pathway, but it does not affect the main conclusion of this study.

UV-Vis absorption and emission spectra of the CdS QDs are shown in as Extended data Fig. 6a. The wavelength of the maximum absorption was observed at 383 nm. The emission spectrum showed a sharp peak at 400 nm attributed to the band gap luminescence, and a broad peak at the wavelength of 550 nm attributed to luminescence from the surface defect levels.

### Synthesis of CdS QDs anchored to SiO_2_ particles

One gram of SiO_2_ particles (10–20 nm, Sigma-Aldrich) dispersed in 50 mL of toluene was heated to 90 °C under nitrogen. One milliliter of (3-mercaptopropyl) trimethoxysilane was injected into the above dispersion. After 24 h stirring, the dispersion was centrifuged to separate the surface-modified SiO_2_ particles. The surface-modified SiO_2_ particles were dried under vacuum and then dispersed in a CdS QDs toluene solution. This dispersion was kept at room temperature for 24 h. The CdS QDs anchored to SiO_2_ particles were separated by centrifugation and dried under vacuum.

The diffuse reflectance and emission spectra of the CdS QDs anchored to SiO_2_ particles are shown in Extended data Fig. 7.

### Preparation of CdS QDs/SiO_2_ thin films

Because the surface of the prepared CdS QDs was capped with oleic acids, the CdS QDs were non-dispersible in water, and the bipyridinium derivatives were soluble in water. To observe the electron transfer between these two materials with different solubilities in water, a film of SiO_2_ particles on which CdS QDs were anchored was prepared on a quartz glass substrate^26^. The films were then immersed in bipyridinium derivative aqueous solutions to measure the photoluminescence.

A semi-transparent SiO_2_ nanoparticle thin film was fabricated by doctor-blading a paste containing 200 mg SiO_2_ powder (10–20 nm, Sigma-Aldrich), 100 mg ethyl cellulose (Tokyo Kasei Kogyo), and 1 mL ethanol onto a quartz glass slide (1 × 4 cm^2^) and then annealed for 1 h at 800 °C. The thin film was immersed in a 3-mercaptopropionic acid aqueous solution (0.1 M) to modify the surface of SiO_2_ nanoparticles to contain a carboxylate group for use as an anchoring group. After drying, the film was immersed in a CdS QDs CH_2_Cl_2_ solution for 1 day and dried. Then, the CdS QDs/SiO_2_ thin film was obtained.

The diffuse reflectance and emission spectra of the CdS QDs/SiO_2_ thin films are shown in Extended data Fig. 6b. These spectra showed the characteristic peaks attributed to CdS QDs. The TEM image of the film (Extended data Fig. 8) showed that CdS QDs with a diameter of 2–3 nm were discretely adsorbed on aggregated SiO_2_ particles with a diameter of 20 nm.

### Synthesis of bipyridinium derivatives

#### N,N’-bis(3-sulfonatopropyl)-4,4′-bipyridinium

PVS was synthesized according to a procedure adapted from Ref. 15, and 0.81 g (5.2 mmol) of 4,4′-bipyridine was added to 1,3-propane sultone (3.25 mL, 37 mmol) heated at 70 °C under nitrogen. Then, acetone (20 mL) was injected into the solution, and the resulting mixture was refluxed for 2 h. After cooling, the white precipitate was filtered, washed with acetone, and dried. Anal. Calcd. for C_16_H_20_N_2_S_2_O_6_·H_2_O: C, 45.92; H, 5.30; N, 6.69. Found: C, 46.47; H, 4.65; N, 6.57. ^1^H NMR (D_2_O, H_2_O in sample as internal standard) δ (ppm): 9.1 (4 H, d, *J* = 7.0 Hz), 8.5 (4 H, d, *J* = 6.7 Hz), 4.8 (4 H, t, *J* = 7.6 Hz), 3.0 (4 H, t, *J* = 7.3 Hz), 2.45 (4 H, tt, *J*_1_ = 7.0 Hz, *J*_2_ = 7.6 Hz)

#### N,N’-bis(3-sulfonatopropyl)-2,2′-bipyridinium

DQS was synthesized according to a procedure adapted from Ref. 19, and 2,2′-bipyridine 0.81 g (5.2 mmol) was added to 1,3-propane sulfone (3.25 mL, 37 mmol) heated at 150 °C under nitrogen. Mesitylene (20 mL) was injected into the resultant solution, and the mixture was refluxed for 1 day. After cooling, the white precipitate was filtered and washed with acetone. The product was then recrystallized from aqueous acetone and dried. Anal. Calcd for C_16_H_20_N_2_S_2_O_6_·2H_2_O: C, 44.03; H, 5.50; N, 6.4. Found: C, 42.82; H, 5.27; N, 5.86. ^1^H NMR (D_2_O, H_2_O in sample as internal standard) δ (ppm): 9.2 (2 H, d, *J* = 6.2 Hz), 8.8 (2 H, dd, *J*_1_ = 8.13 Hz, *J*_2_ = 7.62 Hz), 8.36 (4 H, m), 4.60 (2 H, m), 4.33 (2 H, m), 2.78 (4 H, m), 2.24 (4 H, m).

### Optical measurements

Transmission absorption spectra and diffuse reflectance spectra were determined using a UV-Vis spectrophotometer (Jasco V-570). Emission spectra were obtained using a fluorescence spectrophotometer (Hitachi F-7000).

### Photocatalytic reaction

CdS QDs (0.5 mg) anchored to SiO_2_ particles were dispersed in 2 mM PVS MeOH/H_2_O solution (3 ml, MeOH/H_2_O = 1:14). MeOH enhances dispersing the ability of the CdS QDs anchored to SiO_2_ particles and serves as an electron donating reagent of the photocatalytic reaction. The dispersion was put in the side-arm glass cell with an optical length of 1 cm. The absorption spectrum of the reaction mixture is shown in Extended data Fig. 7. After the glass cell was purged with Ar, it was placed into the ellipsoidal microwave applicator for irradiating the solution in the cell by microwaves. A spectroscopic cryostat (Unisoku CoolSpek UV) was used to maintain the solution temperature in the experiments under conventional heating performed for comparison. The reaction mixture was heated to 52 °C. This temperature of the solution was kept by irradiating microwaves of 1.5 W. The sample was irradiated with a 300 W Xe lamp (Asahi spectra Co. Ltd., MAX-302) equipped with a UV mirror module (λ < 400 nm) for 20 min. During the irradiation with UV, UV-Vis spectroscopic measurements were repeated by every 5 min to determine the quantity of the produced PVS radical anion. The concentration of PVS radial anion was calculated from the absorbance at 602 nm of the reaction solution. The absorption coefficient of PVS radical anion at 602 nm (*ε*_602_), 12800 L mol^−1^ cm^−1^, was used for the calculation^15^. All experiments were performed three times to ensure reproducibility.

### Time-resolved emission decay spectroscopy

We assembled the equipment shown in Extended data Fig. 9 to measure the time-resolved emission decay under microwave irradiation. An ellipsoidal microwave applicator (Chronix Ltd., Extended data Fig. 9b) was introduced into a measuring chamber of a time-resolved emission spectrometer (Hamamatsu Photonics, Quantaurus-Tau C11367-01). Two holes were made at the lateral sides of the ellipsoidal applicator to introduce the excitation light and extract the emission light. A spectroscopic cryostat (Unisoku CoolSpek UV) equipped with a high-temperature control unit (Unisoku CS-AT-HT) was introduced into the time-resolved emission spectrometer to observe the emission decay under conventional heating. CdS QDs/SiO_2_ thin films immersed in a solution in a rectangular four-sided quartz glass cell with an optical length of 1 cm were excited at 365 nm (2 MHz pulse LED). All experiments were performed three times to ensure reproducibility.

### Fitting of time-resolved emission decay spectroscopy

The measured time-resolved emission decay spectra, *I*(*t*), are the convolution of the laser pulse intensity function, *E*(*t*), of the excitation light and emission spectra from samples, *F*(*t*). *I*(*t*) is given as follows:


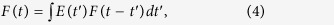


The laser pulse intensity is deconvoluted by this formula. After deconvolution, time-resolved emission spectra are fitted by


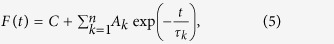


where *C* is a constant from background, *A*_*k*_ is the *k*th pre-exponential factor, and τ_*k*_ is the *k*th emission decay life time. If time-resolved emission spectra are fitted by a biexponential, *n *= 2. If they are fitted by a triexponential, *n *= 3. The *k*th area ratio, *A*_*kNormalized*_, is given by


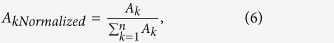


## Additional Information

**How to cite this article**: Kishimoto, F. *et al*. Microwave-enhanced photocatalysis on CdS quantum dots - Evidence of acceleration of photoinduced electron transfer. *Sci. Rep*. **5**, 11308; doi: 10.1038/srep11308 (2015).

## Supplementary Material

Supplementary Information

## Figures and Tables

**Table 1:** Deconvoluted results of time-resolved emission decay profiles of CdS QDs immersed in water or PVS aqueous solution (Time range = 100 ns).

Impregnating fluid	Heat method	*X*^2^	*T*_1_ (ns)	*T*_2_ (ns)	*T*_3_ (ns)	*A*_1 Normalized_	*A*_2 Normalized_	A_3 Normalized_
H_2_O	Conventional heating	1.15	2.8 ± 0.42	31 ± 8.2	—	0.76 ± 0.045	0.24 ± 0.045	—
	Microwave	1.19	2.5 ± 0.34	38 ± 1.4	—	0.82 ± 0.038	0.18 ± 0.028	—
PVSaq	Conventional heating	1.07	0.82 ± 0.31	5.1 ± 0.53	40 ± 9.5	0.60 ± 0.071	0.34 ± 0.058	0.057 ± 0.020
	Microwave	1.06	0.80 ± 0.062	4.3 ± 0.38	34 ± 4.8	0.84 ± 0.0065	0.14 ± 0.0031	0.028 ± 0.0040

**Table 2:** Deconvoluted results of time-resolved emission decay profiles of CdS QDs immersed in water, PVS, or DQS aqueous solutions (Time range = 20 ns).

Impregnating fluid	Heat method	*X*^2^	*T*_av_ (ns)	*T*_1_ (ns)	T_2_ (ns)	*T*_3_ (ns)	*A*_1 Normalized_	*A*_2 Normalized_	*A*_*3 Normalized*_
H2O	Conventional heating	1.12	8.8 ± 1.3	1.8 ± 0.029	35*	—	0.77 ± 0.040	0.23 ± 0.040	—
	Microwae	1.19	9.4 ± 0.99	1.7 ± 0.026	35*	—	0.75 ± 0.034	0.25 ± 0.034	—
PVSaq (2 mM)	Conventional heating	1.05	3.1 ± 0.60	0.61 ± 0.052	3.5 ± 0.15	35*	0.77 ± 0.061	0.17 ± 0.049	0.060 ± 0.012
	Microwave	0.91	1.6 ± 0.087	0.44 ± 0.076	2.9 ± 0.31	35*	0.73 ± 0.025	0.25 ± 0.027	0.015 ± 0.0063
DQSaq (2 mM)	Conventional heating	1.04	4.0 ± 0.68	0.70 ± 0.050	3.7 ± 0.29	35*	0.73 ± 0.042	0.19 ± 0.038	0.085 ± 0.022
	Microwave	1.06	1.7 ± 0.15	0.40 ± 0.021	2.7 ± 0.27	35*	0.76 ± 0.014	0.18 ± 0.017	0.054 ± 0.0084

^*^fixed value.
