# Supplementary material for: Microwave-enhanced photocatalysis on CdS quantum dots - Evidence of acceleration of photoinduced electron transfer
Source: Sci Rep. 2015 Jun 17;5:11308. doi: 10.1038/srep11308 (PMC4469968; doi:10.1038/srep11308)
Supplement: Supplementary Information [file srep11308-s1.pdf]

Supplementary Information

Microwave-enhanced photocatalysis on CdS quantum dots – Evidence of acceleration of photoinduced electron transfer

Authors: Fuminao Kishimoto[1], Takashi Imai[1], Satoshi Fujii[1,2], Dai Mochizuki[1], Masato M. Maitani[1], Eiichi Suzuki[1], Yuji Wada[1]*.

Affiliations:

[1] Department of Applied Chemistry, Tokyo Institute of Technology, 2-12-10-E4-3 Ookayama, Meguro, Tokyo 152-8551, Japan.

[2] Knowledge-intensive Collaborative Research Center, Chiba University, 1-33 Yayoi-cho, Inage-ku, Chiba 263-8522, Japan.

*Correspondence to: E-mail: yuji-w@apc.titech.ac.jp (Y. W.)

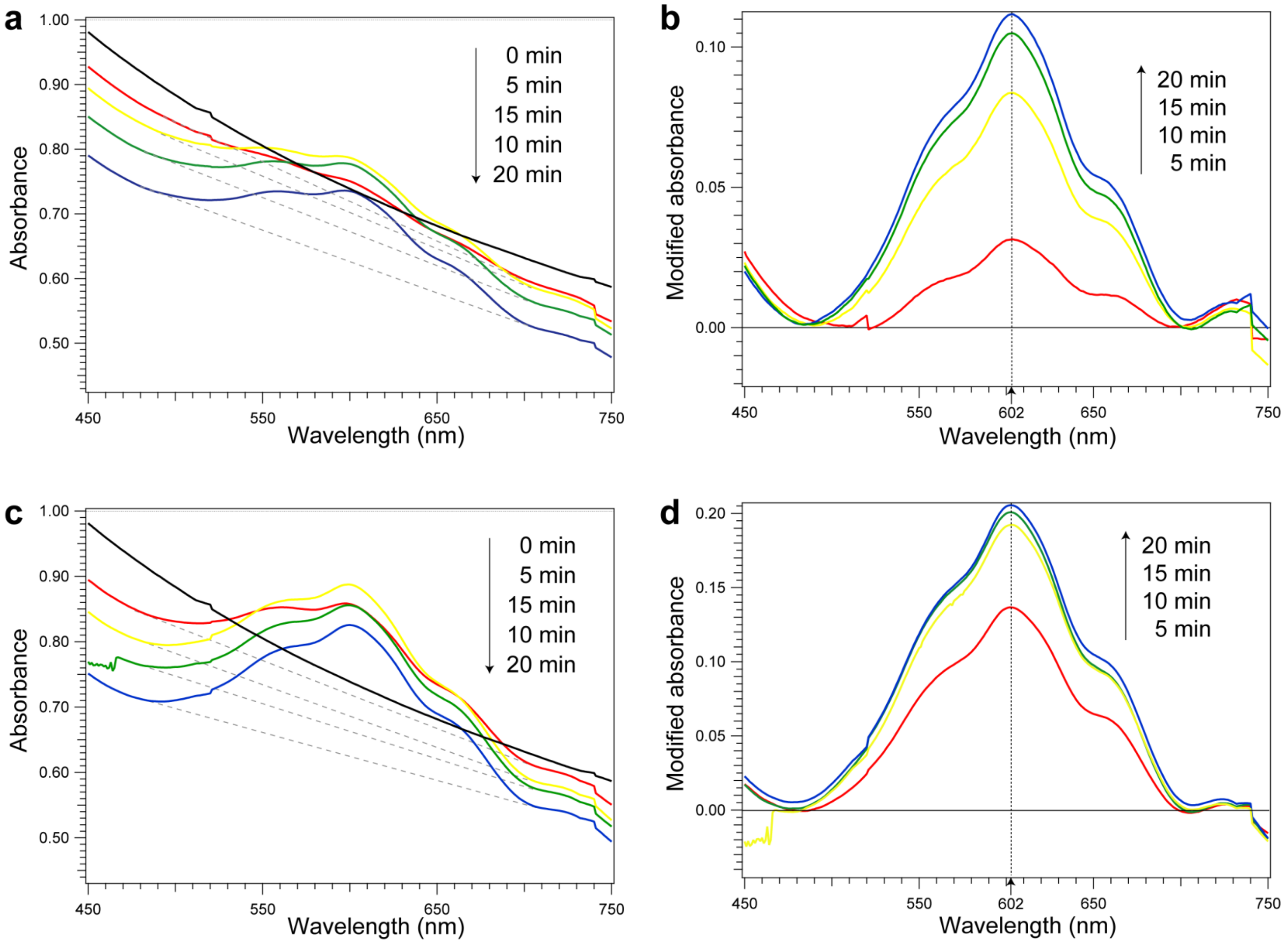

Extended data Fig. 1| Time variation absorption spectra of the reaction solutions for photocatalytic reduction of PVS. (a) Raw absorption spectra under conventional heating. Because the reaction mixture was not stirred, the scattering caused by dispersed SiO_2 particles with anchored CdS QDs anchored to SiO_2 was largest at the beginning of the reaction and diminishing with the reaction time. The dotted lines are the interpolated baselines. **(b)** Corrected absorption spectra under conventional heating by subtracting the baselines. **(c)** Raw absorption spectra under microwaves. **(d)** Corrected absorption spectra under microwaves by subtracting the baselines.

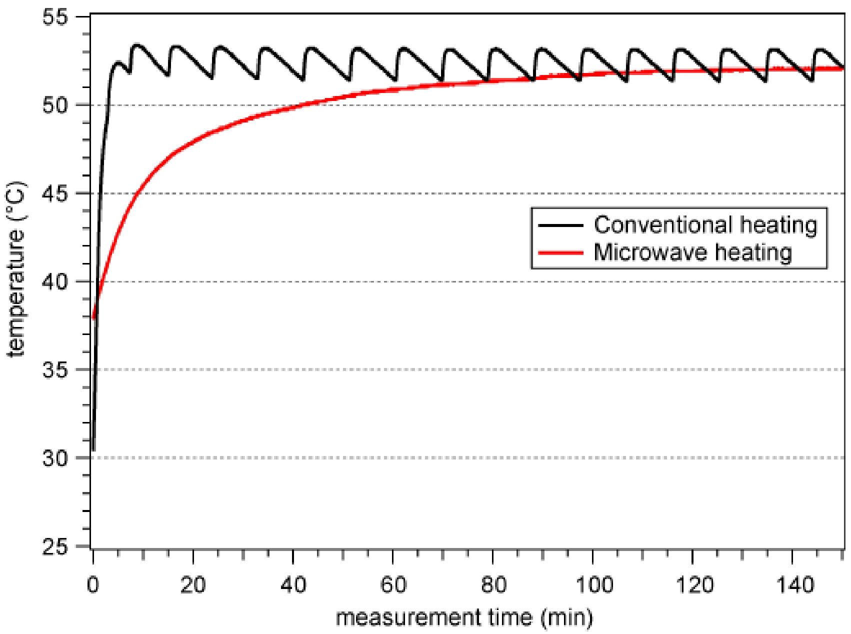

Extended data Fig. 2| Temperature profiles of PVS aqueous solution during the measurements of time-resolved emission profiles. Red: microwave irradiated at 1.0 W; Black: Conventional heating (UNISOKU CoolSpek UV).

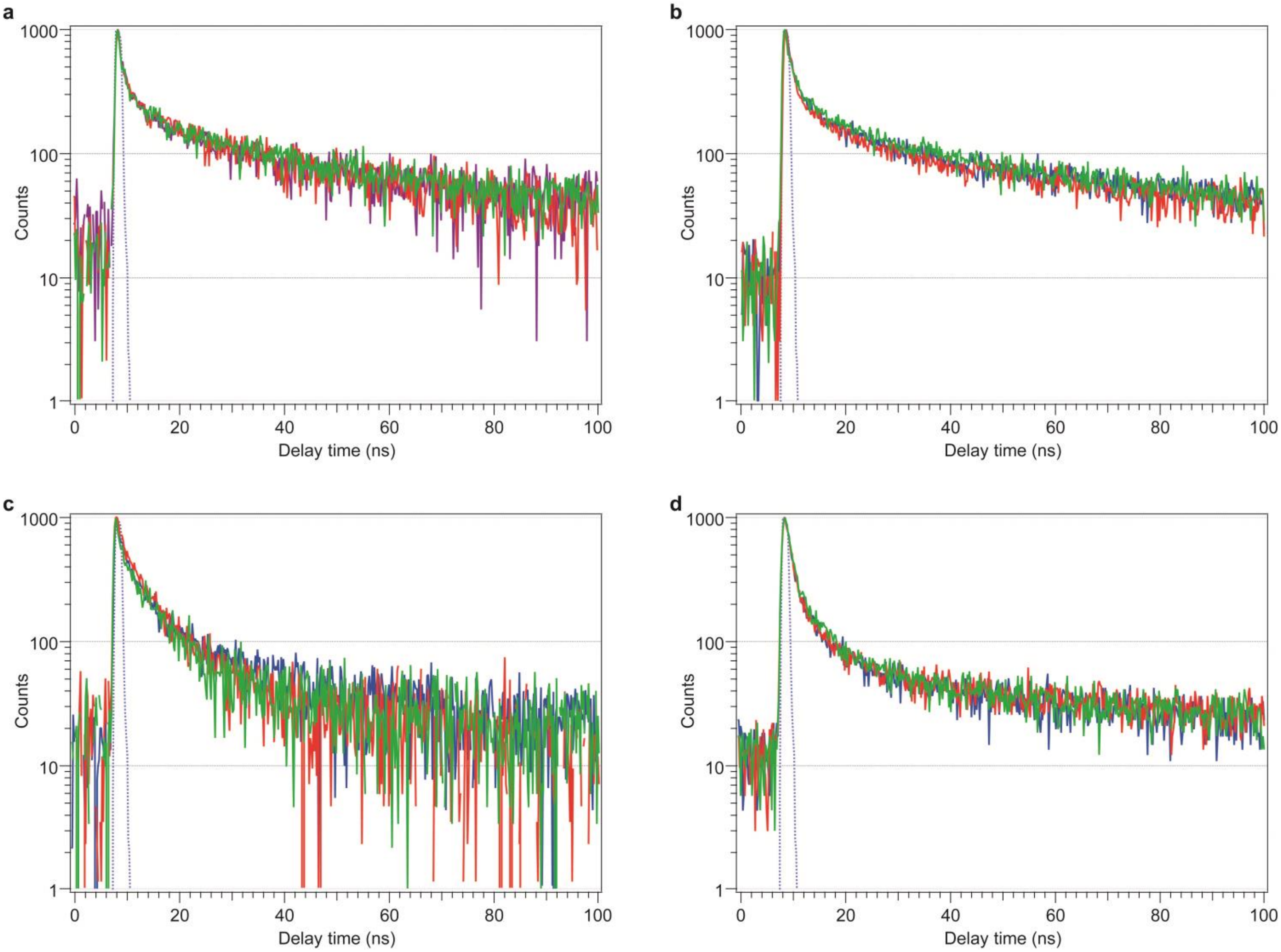

Extended data Fig. 3| All profiles of time-resolved emission decay of CdS QDs/SiO_2. (time range = 100 ns)

The emission decay profiles were measured three times at every experimental condition. (**a**) Time-resolved emission decay profiles of CdS QDs/SiO_2 immersed water under conventional heating. (**b**) Time-resolved emission decay profiles of CdS QDs/SiO_2 immersed in water under microwave heating. (**c**) Time-resolved emission decay profiles of CdS QDs/SiO_2 immersed in PVS aqueous solution under conventional heating. (**d**) Time-resolved emission decay profiles of CdS QDs/SiO_2 immersed in PVS aqueous solution under microwave irradiation.

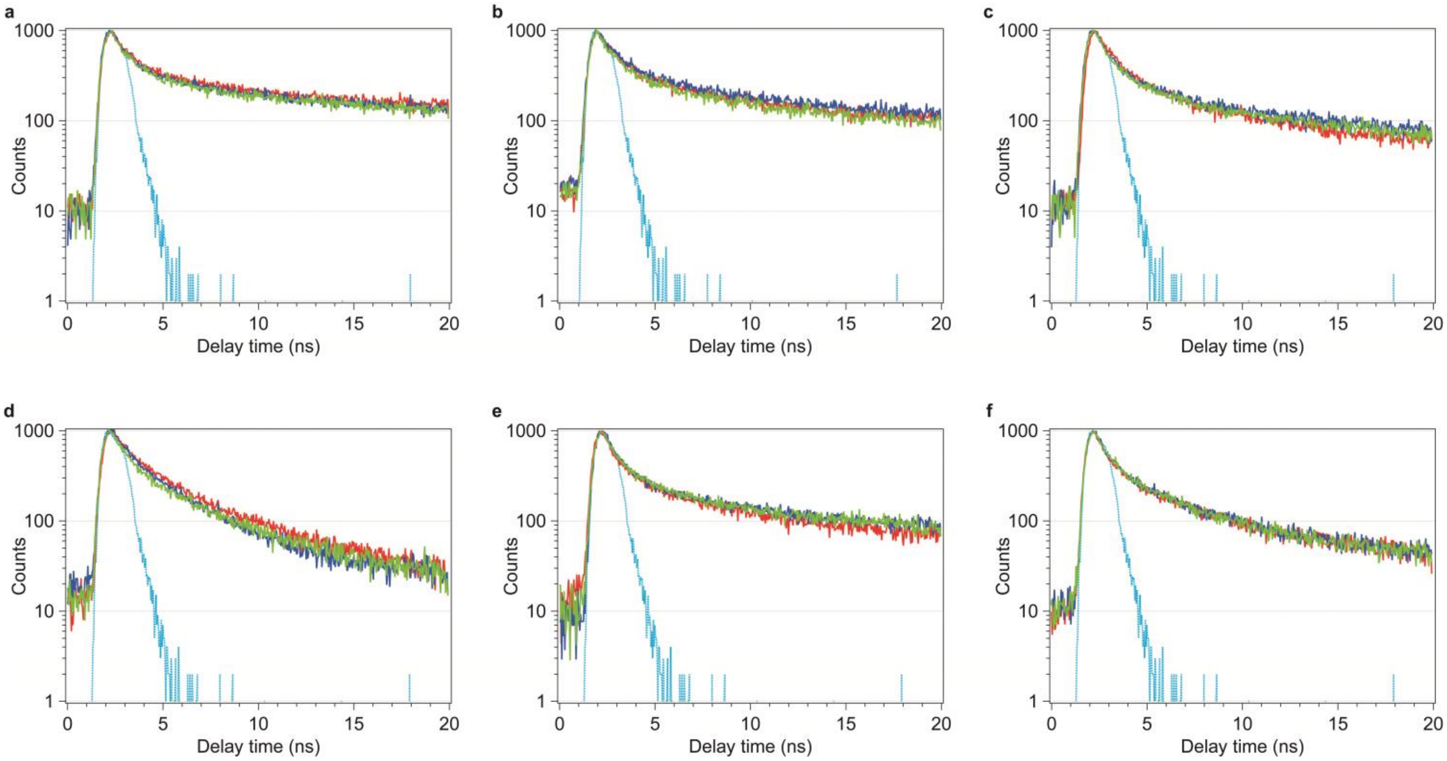

Extended data Fig. 4| All profiles of time-resolved emission decay of dispersed CdS QDs/SiO_2. (time range = 20 ns) The emission decay profiles were measured three times at every experimental condition. (**a**) Time-resolved emission decay profiles of CdS QDs/SiO_2 immersed in water under conventional heating. (**b**) Time-resolved emission decay profiles of CdS QDs/SiO_2 immersed in water under microwave heating. (**c**) Time-resolved emission decay profiles of CdS QDs/SiO_2 immersed in PVS aqueous solution under conventional heating. (**d**) Time-resolved emission decay profiles of CdS QDs/SiO_2 immersed in PVS aqueous solution under microwave irradiation. (**e**) Time-resolved emission decay profiles of CdS QDs/SiO_2 immersed in DQS aqueous solution under conventional heating. (**f**) Time-resolved emission decay profiles of CdS QDs/SiO_2 immersed in DQS aqueous solution under microwave irradiation.

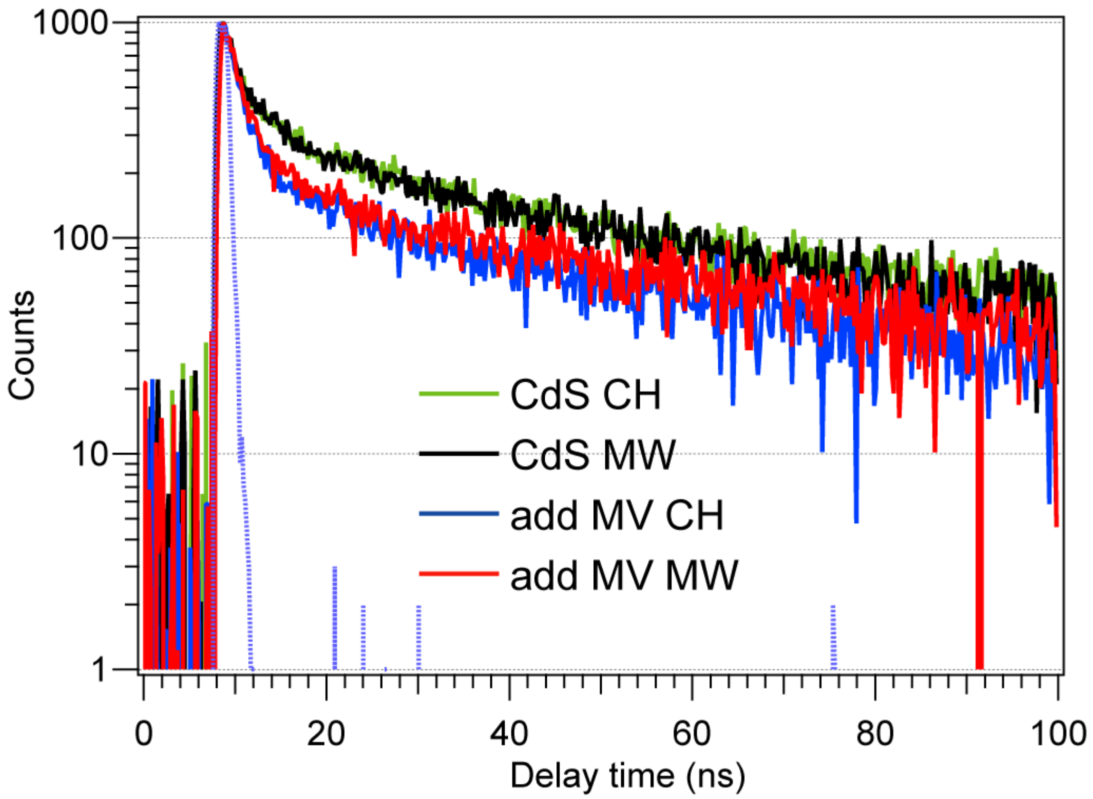

Extended data Fig. 5| Time-resolved emission decay profiles of dispersed CdS QDs. Methyl viologen (MV; Sigma-Aldrich) methanol solution was added to a dispersion of CdS QDs in toluene.

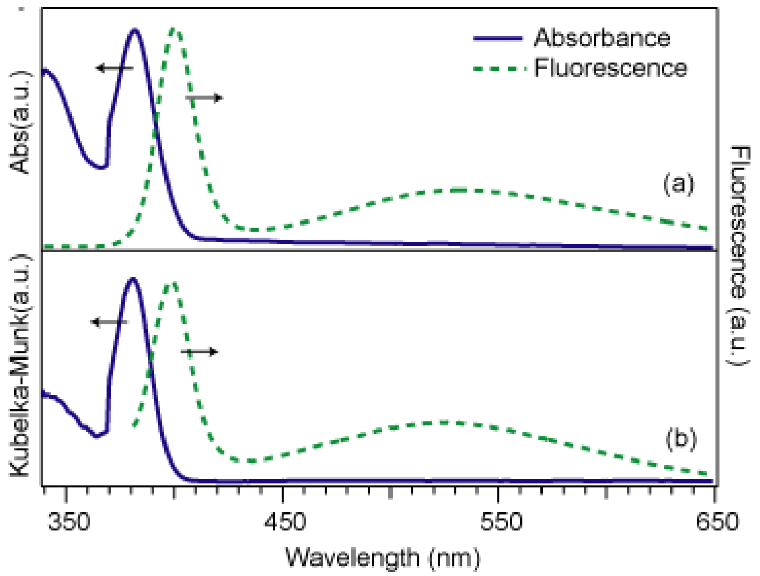

Extended data Fig. 6| UV-vis absorption and emission spectra. (a) Absorption (blue solid line) and emission spectra (green dotted line) of the CdS QDs dispersed in CH_2Cl_2. (b) Diffuse reflectance (blue solid line) and emission spectra (green dotted line) of CdS QDs/SiO_2 thin films.

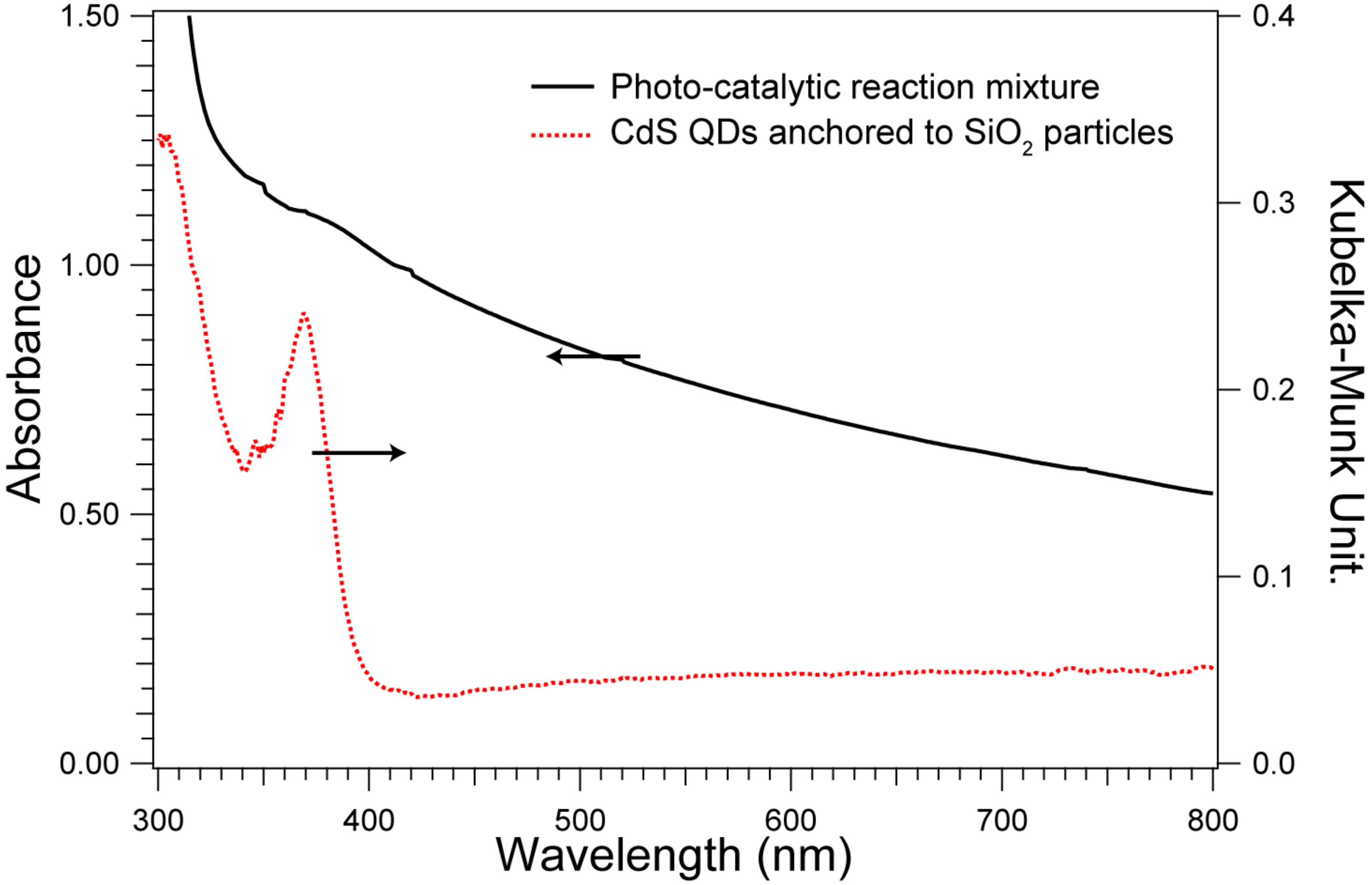

Extended data Fig. 7| Absorption spectra of CdS QDs anchored to SiO_2 particles (dotted line, diffuse reflectance mode) and the photocatalytic reaction mixture containing 2 mM PVS, H_2O, MeOH and CdS QDs anchored to SiO_2 (solid line, transmission mode). The diffuse reflectance spectrum of the CdS QDs anchored to SiO_2 particles shows a peak at ca. 370 nm attributed to CdS QDs. The spectrum of the reaction mixture shows a wide-range scattering by SiO_2 particles over the visible region.

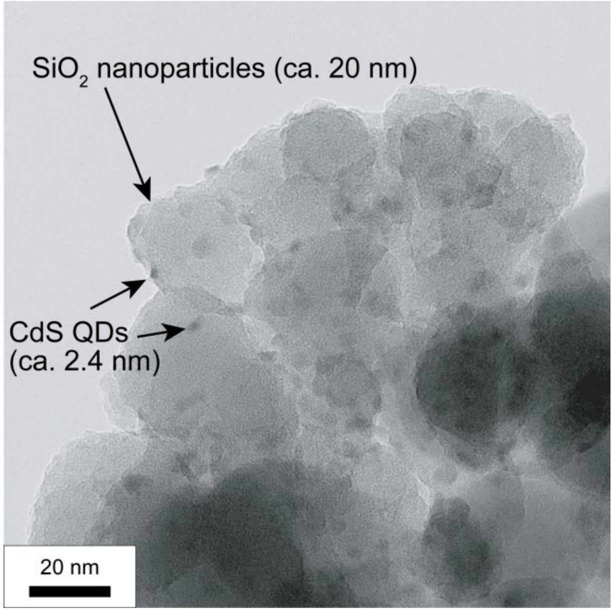

Extended data Fig. 8| TEM image of CdS QDs/SiO$_2$ thin films. CdS QDs were discretely adsorbed on aggregated SiO$_2$ nanoparticles.

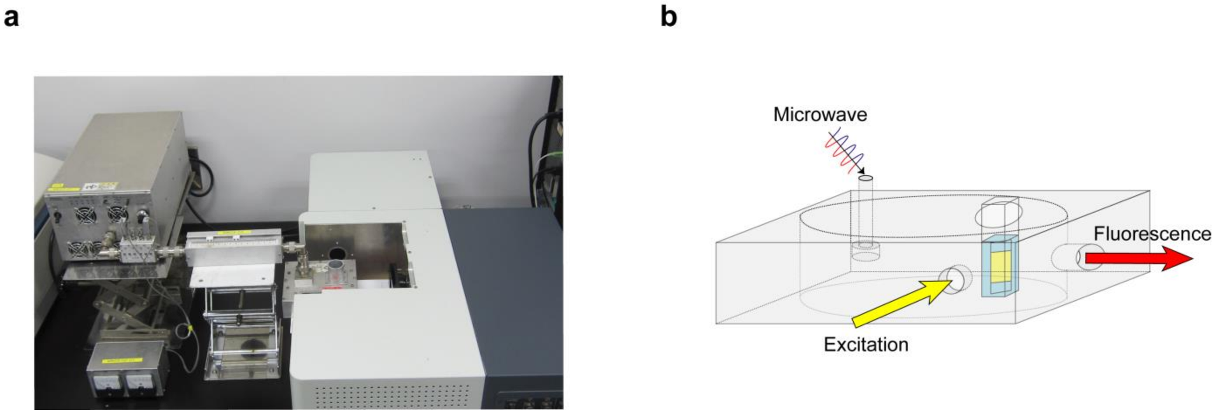

Extended data Fig. 9| Equipment for measuring time-resolved emission decay profiles under microwave irradiation. (**a**) Photograph of a spectrometer installed with a microwave applicator. An ellipsoid microwave applicator (CHRONIX Ltd.,) was introduced into a measuring chamber of a time-resolved emission spectrometer. (**b**) Graphic of an ellipsoid microwave applicator. A microwave antenna was located at one focus point and a sample was placed at the other focus point. Two holes were drilled at the lateral faces for introducing the excitation light and extracting the emission light.

Extended data Table 1| All Deconvoluted results of time-resolved emission decay profiles of CdS QDs immersed in neat water and PVS aqueous solution (Time range = 100 ns)

Impregnating fluid	Heat method	Entry	χ^2	τ_1 (ns)	τ_2 (ns)	τ_3 (ns)	$A_{1\ Normalized}$	$A_{2\ Normalized}$	$A_{3\ Normalized}$
H_2O	Conventional heating	1	0.979	2.70	23.0	-	0.722	0.278	-
		2	1.25	3.22	39.4	-	0.755	0.245	-
		3	1.22	2.38	31.0	-	0.811	0.189	-
		ave.	1.15	2.8 ± 0.42	31 ± 8.2	-	0.76 ± 0.045	0.24 ± 0.045	-
	Microwave	1	1.12	2.49	38.1	-	0.804	0.196	-
		2	1.16	2.87	38.7	-	0.788	0.212	-
		3	1.28	2.19	36.1	-	0.861	0.139	-
		ave.	1.19	2.5 ± 0.34	38 ± 1.4	-	0.82 ± 0.038	0.18 ± 0.028	-
PVSaq	Conventional heating	1	0.995	1.11	4.58	32.2	0.580	0.343	0.0765
		2	1.02	0.844	5.12	38.3	0.544	0.398	0.0584
		3	1.19	0.493	5.64	50.9	0.681	0.282	0.0372
		ave.	1.07	0.82 ± 0.31	5.1 ± 0.53	40 ± 9.5	0.60 ± 0.071	0.34 ± 0.058	0.057 ± 0.020
	Microwave	1	0.987	0.876	4.68	38.6	0.843	0.132	0.0255
		2	1.09	0.770	4.29	29.2	0.837	0.137	0.0263
		3	1.10	0.767	3.92	35.6	0.830	0.137	0.0328
		ave.	1.06	0.80 ± 0.062	4.3 ± 0.38	34 ± 4.8	0.84 ± 0.0065	0.14 ± 0.0031	0.028 ± 0.0040

Extended data Table 2| Deconvoluted results of time-resolved emission decay profiles of CdS QDs immersed in PVS aqueous solution by biexponential function (Time range = 100 ns)

Impregnating fluid	Heat method	Entry	χ^2	τ_1 (ns)	τ_2 (ns)	$A_{1\ Normalized}$	$A_{2\ Normalized}$
PVS	Conventional heating	1	1.44	2.34	20.1	0.848	0.152
		2	1.26	3.44	28.2	0.906	0.0955
		3	1.38	4.12	32.7	0.914	0.0859
		ave.	1.36	3.3 ± 0.89	27 ± 6.4	0.89 ± 0.36	0.11 ± 0.036
	Microwave	1	1.49	1.65	21.6	0.941	0.0588
		2	1.34	1.27	13.8	0.933	0.0672
		3	1.43	1.63	25.6	0.985	0.0150
		ave.	1.42	1.5 ± 0.21	20 ± 6.0	0.97 ± 0.0828	0.03 ± 0.028

Extended data Table 3| All dconvoluted results of time-resolved emission decay profiles of CdS QDs immersed in neat water, PVS aqueous solution and DQS aqueous solution (Time range = 20 ns)

Impregnating fluid	Heat method	Entry	χ^2	τ_{av} (ns)	τ_1 (ns)	τ_2 (ns)	τ_3 (ns)	$A_{1\ Normalized}$	$A_{2\ Normalized}$	$A_{3\ Normalized}$
H_2O	Conventional heating	1	1.11	2.70	1.80	35*	-	0.730	0.270	-
		2	1.17	3.22	1.75	35*	-	0.783	0.217	-
		3	1.09	2.38	1.75	35*	-	0.809	0.191	-
		ave.	1.12	8.8 ± 1.3	1.8 ± 0.029	35*	-	0.77 ± 0.040	0.23 ± 0.040	-
	Microwave	1	1.18	9.74	1.96	35*	-	0.749	0.251	-
		2	1.17	10.2	1.44	35*	-	0.721	0.279	-
		3	1.21	8.34	1.76	35*	-	0.789	0.211	-
		ave.	1.19	9.4 ± 0.99	1.7 ± 0.026	35*	-	0.75 ± 0.034	0.25 ± 0.034	-
PVSaq	Conventional heating	1	1.16	2.56	0.569	3.33	35*	0.817	0.133	0.0500
		2	0.95	2.87	0.587	3.57	35*	0.790	0.153	0.0565
		3	1.05	3.72	0.666	3.62	35*	0.670	0.226	0.0739
		ave.	1.05	3.1 ± 0.60	0.61 ± 0.052	3.5 ± 0.15	35*	0.77 ± 0.061	0.17 ± 0.049	0.060 ± 0.012
	Microwave	1	0.792	1.46	0.528	2.97	35*	0.718	0.273	0.00805
		2	1.02	1.59	0.407	3.18	35*	0.759	0.224	0.0173
		3	0.925	1.62	0.387	2.57	35*	0.715	0.265	0.0201
		ave.	0.91	1.6 ± 0.087	0.44 ± 0.076	2.9 ± 0.31	35*	0.73 ± 0.025	0.25 ± 0.027	0.015 ± 0.0063
DQSaq	Conventional heating	1	0.959	4.20	0.674	3.56	35*	0.758	0.146	0.0961
		2	1.09	4.58	0.761	3.58	35*	0.679	0.222	0.0991
		3	1.07	3.27	0.675	4.07	35*	0.746	0.194	0.0598
		ave.	1.04	4.0 ± 0.68	0.70 ± 0.050	3.7 ± 0.29	35*	0.73 ± 0.042	0.19 ± 0.038	0.085 ± 0.022
	Microwave	1	1.06	1.54	0.405	2.45	12.7	0.760	0.177	0.0631
		2	1.05	1.83	0.426	2.99	19.1	0.750	0.203	0.0471
		3	1.07	1.63	0.385	2.75	16.8	0.777	0.172	0.0509
		ave.	1.06	1.7 ± 0.15	0.40 ± 0.021	2.7 ± 0.27	35*	0.76 ± 0.014	0.18 ± 0.017	0.054 ± 0.0084

*fixed value
